# Genetic Architecture of Cereal Leaf Beetle Resistance in Wheat

**DOI:** 10.3390/plants9091117

**Published:** 2020-08-28

**Authors:** Tobias Würschum, Willmar L. Leiser, Simon M. Langer, Matthew R. Tucker, Thomas Miedaner

**Affiliations:** 1 Institute of Plant Breeding, Seed Science and Population Genetics, University of Hohenheim, 70593 Stuttgart, Germany; 2 State Plant Breeding Institute, University of Hohenheim, 70593 Stuttgart, Germany; willmar_leiser@uni-hohenheim.de (W.L.L.); simon-martin.langer@basf.com (S.M.L.); thomas.miedaner@uni-hohenheim.de (T.M.); 3 BASF Agricultural Solutions GmbH, 67063 Ludwigshafen, Germany; 4 School of Agriculture, Food and Wine, Waite Campus, University of Adelaide, 5064 Urrbrae, Australia

**Keywords:** cereal leaf beetle, wheat, heading time, *Ppd-D1*, glaucousness, waxiness

## Abstract

Wheat production can be severely damaged by endemic and invasive insect pests. Here, we investigated resistance to cereal leaf beetle in a panel of 876 winter wheat cultivars, and dissected the genetic architecture underlying this insect resistance by association mapping. We observed an effect of heading date on cereal leaf beetle infestation, with earlier heading cultivars being more heavily infested. Flag leaf glaucousness was also found to be correlated with resistance. In line with the strong effect of heading time, we identified *Ppd-D1* as a major quantitative trait locus (QTL), explaining 35% of the genotypic variance of cereal leaf beetle resistance. The other identified putative QTL explained much less of the genotypic variance, suggesting a genetic architecture with many small-effect QTL, which was corroborated by a genomic prediction approach. Collectively, our results add to our understanding of the genetic control underlying insect resistances in small-grain cereals.

## 1. Introduction

Wheat (*Triticum aestivum* L.) is one of the most important food and feed crops worldwide. The huge geographical distribution of wheat cultivation also entails a large array of environmental conditions to which it is exposed. Besides abiotic stresses, various biotic stresses can severely affect wheat yield. Among them, fungal diseases are of great importance, but insect pests also cause substantial damage to wheat production in many traditional wheat-producing areas. While pesticides can be used to control disease, the employment of genetic resistance is the most economic and environmentally friendly way of protecting crop production. Especially with the banning of certain pesticides or entire classes thereof, as well as public trends towards reduced or even no pesticide use, the importance of developing resistant cultivars increases. A prerequisite for an efficient utilization of pest resistances in breeding is, however, a detailed understanding of the genetics underlying the resistance mechanism(s).

**Figure 1:**
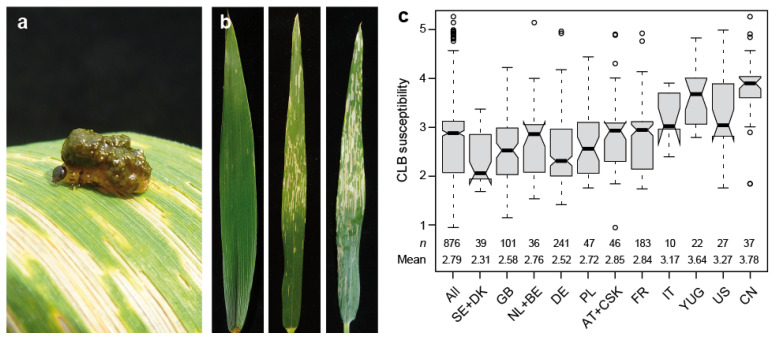
Cereal leaf beetle (CLB) resistance in wheat. (**a**) Cereal leaf beetle larvae on a wheat flag leaf, (**b**) Examples of flag leaves with varying degrees of susceptibility (score of 1, 3 and 4 from left to right), (**c**) Boxplots showing susceptibility dependent on the cultivars’ country of origin. AT, Austria; BE, Belgium; CN, China; CSK, former Czechoslovakia; DE, Germany; DK, Denmark; FR, France; GB, Great Britain; IT, Italy; NL, The Netherlands; PL, Poland; SE, Sweden; US, United States of America; YUG, former Yugoslavia, Serbia, Croatia.

Among the insect pests threatening wheat production, the Hessian fly (*Mayetiola destructor* Say), Sunn pest (*Eurygaster integriceps* Puton), Russian wheat aphid (*Diuraphis noxia* Kurdjumov), wheat stem saw fly (*Cephus cinctus* Norton), aphids (*Sitobion* = *Macrosiphum avenae* Fabr., *Rhopalosiphum padi* L., *Metopolophium dirhodum* Walk.), fruit fly (*Oscinella frit*), wheat blossom midges (*Contarinia tritici*, *Sitodiplosis mosellana*) and cereal leaf beetle (*Oulema* spp., with *O. melanopus* L. being dominant) can cause severe damage and thus annual economic losses in the regions where they occur. Cereal leaf beetle (CLB; Coleoptera: Chrysomelidae) is a major pest of small grains that is native to Europe and Asia, but also occurs in the United States, where it was accidentally introduced around 1950 and first reported in the early 1960s [1,2]. It infests a large range of wild and cultivated grasses, including barley, rye, oats, and wheat. The beetle produces one primary generation per year, the adults mating in spring and the eggs being deposited along the midvein on the upper leaf surface [2]. It is the larvae, particularly older larvae, that do the most damage to the crops, as they eat long strips of the upper leaf surface and parenchyma tissue, thus skeletonizing the leaves and reducing the plants’ photosynthetic potential and thereby yield [1]. The larvae typically smear a black globule of mucus and excrement on their backs, as a defense mechanism, to mask their yellowish color and to deter predators (Figure 1a). Damage due to cereal leaf beetle infestation is usually highly variable, depending on infestation levels, field characteristics and environmental conditions, with maximum losses estimated at approximately 40% [1]. Herbert et al. [3] reported that in Virginia (USA) commercial wheat fields, the yield loss averages around 15% if the pest is left untreated. However, there are several insecticides registered for small grains (e.g., synthetic pyrethroids), that allow an efficient control of cereal leaf beetle at relatively low cost.

In small-grain cereals, insect resistances have received far less attention in recent years and decades than fungal diseases. This is in part attributable to their generally lower economic importance, but also to the difficulties in phenotypically assessing them, particularly under field conditions. For cereal leaf beetle, Papp and Mesterházy [4] performed resistance tests with 26 winter wheat genotypes, comparing infested and non-infested control plots covered by insect nets, and observed highly significant genotypic differences. The only study on the genetic control underlying cereal leaf beetle resistance was reported by Joukhadar et al. [5], who used an association mapping approach with 134 wheat genotypes and natural infestation. This resulted in the identification of two putative quantitative trait loci (QTL) on chromosomes 3B and 7D, explaining 43 and 33% of the phenotypic variance, respectively. Generally, besides resistance mechanisms, certain plant characteristics may also contribute to insect resistance, for example leaf traits such as glaucousness, or phenological traits such as heading time. The latter might provide an escape mechanism, as is known for early heading and drought stress.

The aim of this study was to improve our understanding of the genetic control underlying resistance to cereal leaf beetle infestation. To this end, we employed a large panel of 876 winter wheat cultivars that were phenotyped for resistance in the field and genotyped with genome-wide markers. In particular, our objectives were to (I) evaluate the distribution of the phenotypic resistance values and their relationship with the cultivars’ country of origin, (II) investigate the correlation with heading time and flag leaf glaucousness, (III) perform genome-wide association mapping, and (IV) complement the picture of the genetic architecture underlying cereal leaf beetle resistance by genomic prediction.

## 2. Materials and Methods 

### 2.1. Plant Material and Experimental Design

A panel of 876 soft winter wheat (*Triticum aestivum* L.) cultivars was used for this study, representing a subset of the panel of 1110 cultivars described previously [6,7]. This panel includes wheat cultivars of worldwide origin that were released during the past decades, but with the majority originating from Europe. The cultivars were grown in observation plots of two rows and 1.25 m length at the location Oberer Lindenhof (OLI, 48°28′25.5′′ N, 9°18′17.9′′ E, 700 m asl) in 2013, in a partially replicated design, with a replication rate of 1.25 [8]. In addition, data from a second location, Hohenheim (48°42′5′′ N, 9°12′5′′ E, 400 m asl), were available. At this location, the repeatability was lower at 0.25, and thus, these data were only used for validation. Resistance to cereal leaf beetle was scored on flag leaves on a scale from 1 to 5, as follows: 1, no damage; 2, few lines of feeding damage on the upper leaf side; 3, several lines of feeding damage; 4, 50–70% of the flag leaf showing feeding damage; 5, more than 70% of the flag leaf showing feeding damage. Flag leaf glaucousness was scored on a scale of 1 to 6, as described previously [9]. Briefly, a score of 1 was given when no glaucousness was visible on both sides of the flag leaves, 2 for a light glaucousness on the abaxial flag leaf side, 3 for a strong glaucousness on the proximal half of the abaxial leaf surface, 4 for a strong glaucousness on the entire abaxial leaf surface, 5 for a strong glaucousness on the entire abaxial and half of the adaxial leaf surface, and 6 for fully glaucous flag leaves.

Analysis of the phenotypic data was completed using a model adjusting for replication and block effects, and in addition, spatial modeling was applied by fitting a first order autoregressive variance model for both rows and columns (AR1 ⊗ AR1). Best linear unbiased estimates (BLUEs) were estimated assuming fixed effects for the genotype, while the variance component estimates were derived from a full random model. Heritability (*h*^2^) was estimated following the approach suggested by Piepho and Möhring [10]. All statistical analyses were performed using the statistical software R [11] and ASReml-R 3.0 [12].

### 2.2. Genotypic Analysis and Association Mapping

All lines were genotyped by genotyping-by-sequencing (GBS) at Diversity Arrays Technology (Yarralumla, Australia), using the Wheat GBS 1.0 assay (DArTseq). Markers with a minor allele frequency <0.05 were removed, resulting in a total of 23,720 markers for which a map position was available [13]. The CloneIDs of the silico DArT markers were given a ‘D’ and that of the SNP markers a ‘S’ prefix. Genotyping of *Ppd-D1* was carried out as described by Beales et al. [14]

For association mapping, an additive genetic model was chosen and mapping was carried out with a mixed model, incorporating a kinship matrix, as described previously [7]. To control for multiple testing, a Bonferroni-corrected threshold of *p* < 0.05 was applied. In addition, a less stringent threshold of *p* < 0.0005, suitable for exploratory analyses, was used. The total proportion of genotypic variance (*p_G_*) explained by the detected putative QTL was calculated by fitting all significantly associated markers simultaneously in a linear model, in the order of the strength of their association. The ratio *p_G_* = $R_{adj}^{2}$/*h*^2^, where $R_{adj}^{2}$ refers to the adjusted $R^{2}$ from the linear model and *h*^2^ to the heritability of the trait, yielded the proportion of genotypic variance [15]. The *p_G_* values of individual putative QTL were accordingly derived from their sums of squares (*SS_QTL_*) in this linear model. The allele substitution (α) effects were derived as the regression coefficient from models, with only the marker under consideration.

## 3. Results

This study was based on 876 winter wheat cultivars, and revealed a highly significant (*p* < 0.01) genotypic variance for resistance to cereal leaf beetle evaluated under field conditions (Table S1). Cereal leaf beetle infestation, scored from 1 (no damage on the flag leaf) to 5 (more than 75% of the flag leaf damaged), was found throughout the field, with only minor trends discernible (Figure 1 and Figure S1). The heritability increased from 0.53 for a model adjusting for replication and block effects, to 0.63 when spatial modeling was applied to account for field trends. The trait values (BLUEs) were found to range from 0.95 to 5.26, with a mean of 2.79, thus covering the full spectrum from resistant to highly susceptible. The analysis of susceptibility dependent on the cultivars’ country of origin revealed a substantially higher susceptibility in cultivars originating from Italy, the former Yugoslavia, the US and China (Figure 1c).

**Figure 2:**
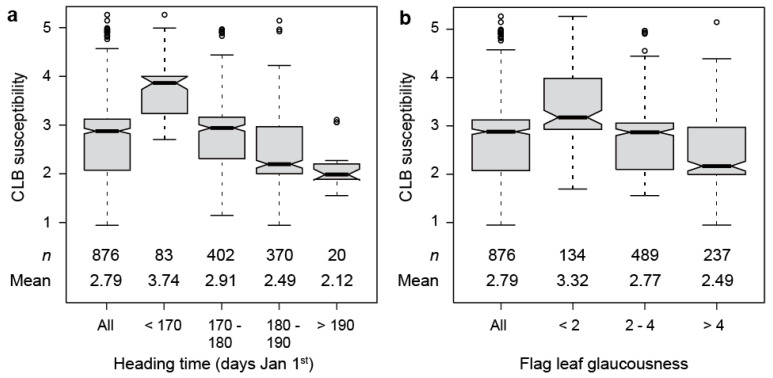
Boxplots showing the association between cereal leaf beetle (CLB) susceptibility and (**a**) heading time and (**b**) flag leaf glaucousness (scored on a 1–6 scale, with 1 being no glaucousness on the abaxial and adaxial sides of the flag leaves, and 6 being fully glaucous flag leaves).

We next investigated the association between cereal leaf beetle susceptibility and heading time, as well as flag leaf glaucousness (Figure 2). This analysis revealed a higher susceptibility of the earlier heading cultivars than the later heading ones, with a correlation of −0.50 (*p* < 0.001). Likewise, we observed a trend towards higher resistance with increasing flag leaf glaucousness, the correlation being −0.35 (*p* < 0.001). This correlation was observed in genotypes homozygous for either the later heading *Ppd-D1b* (−0.23, *p* < 0.001), or the earlier heading *Ppd-D1a* (−0.27, *p* < 0.001) allele. The correlation between cereal leaf beetle susceptibility and plant height was −0.21 (*p* < 0.001). Similar trends were observed at the second location (Figure S2a,b). 

**Figure 3:**
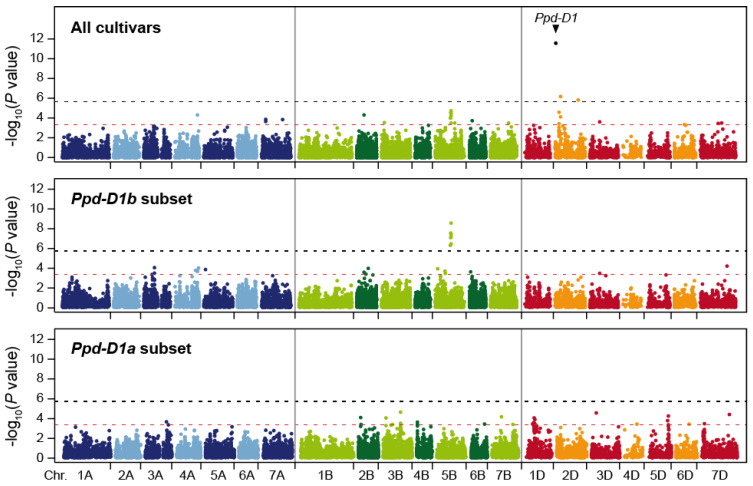
Manhattan plots showing the results from the genome-wide scan for cereal leaf beetle resistance in the entire panel, as well as in the *Ppd-D1b* (photoperiod sensitive) and *Ppd-D1a* (photoperiod insensitive) subsets. The black dashed horizontal line indicates the significance threshold (Bonferroni-corrected *p* < 0.05), and the red dashed horizontal line the exploratory significance threshold (*p* < 0.0005).

All cultivars were genotyped with 23,720 mapped, genome-wide markers, and in addition for the major photoperiod sensitivity locus *Ppd-D1* on chromosome 2D. The genome-wide scan yielded two markers significantly associated with cereal leaf beetle susceptibility at a Bonferroni-corrected significance level of *p* < 0.05 (i.e., *p* < 2.1 × 10^−6^) (Figure 3 and Figure S3). These two markers were both located on chromosome 2D at 58 and 245 cM. In addition, we found *Ppd-D1* to be highly significantly (*p* = 2.7 × 10^−12^) associated with cereal leaf beetle resistance. The marker with the highest linkage disequilibrium with *Ppd-D1* is located at 51 cM, indicating that the peak in that chromosomal region, including the significantly associated marker, represents the association of *Ppd-D1*. Employing a less stringent, exploratory significance threshold of *p* < 0.0005 identified additional putative QTL on several chromosomes (Figure 3, Table 1). 

**Figure 4:**
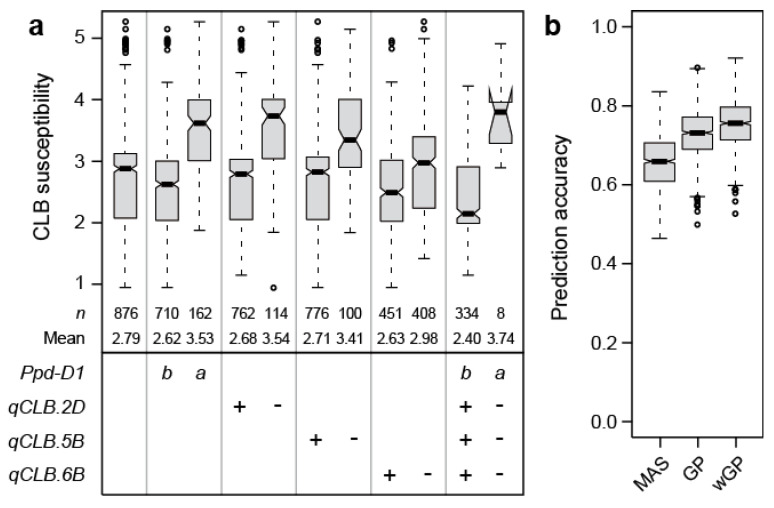
(**a**) Boxplots showing the effects of individual QTL (qCLB), explaining more than 4% of the genotypic variance, as well as the combination of their resistant (+) or susceptible (-) alleles on cereal leaf beetle (CLB) susceptibility. For *Ppd-D1*, *b* refers to the wild-type allele and *a* to the photoperiod-insensitive allele, (**b**) Boxplots showing the prediction accuracy for marker-assisted selection (MAS), based on the two markers significant at the Bonferroni corrected threshold (*Ppd-D1*, *qCLB.2D*), compared to genomic prediction (GP), or a weighted genomic prediction (wGP) that incorporates the two markers as fixed effects.

Jointly, these putative QTL explained 62.2% of the genotypic variance. By far, the highest proportion of genotypic variance was attributable to *Ppd-D1* with 35.3%. All other markers explained less than 10% of the genotypic variance, the next highest contribution coming from the markers on chromosomes 5B, 2D, and 6B, explaining 6.5, 6.2, and 4.0%, respectively. The strongest allele substitution effect was found for *Ppd-D1* with 0.46, while that of the other three loci ranged from 0.17 to 0.43 (Table 1, Figure 4a). While less pronounced, the significant effect of *Ppd-D1* on cereal leaf beetle infestation was confirmed at the second location (Figure S2c).

Owing to the strong effect of *Ppd-D1* on cereal leaf beetle resistance, we also performed the genome-wide association scan in subsets of plants homozygous for either the photoperiod sensitive *Ppd-D1b* (*n* = 710) or the photoperiod insensitive *Ppd-D1a* (*n* = 162) allele. This revealed some overlapping patterns, but also differences in the genetic architecture in both allelic groups. For example, the putative QTL on chromosome 5B stems from the photoperiod sensitive *Ppd-D1b* group, and does not appear to be present in the *Ppd-D1a* cultivars (Figure 3, Table S2).

To complement the picture of the genetic architecture of cereal leaf beetle resistance, we employed a genomic prediction approach. The prediction accuracy of the two markers significant at the Bonferroni corrected threshold (i.e., *Ppd-D1*, D1104237) averaged 0.64, whereas the genome-wide approach averaged 0.71 and the weighted genome-wide approach with the two markers included as fixed effects averaged 0.74 (Figure 4b).

## 4. Discussion

Cereal leaf beetle is a wheat pest that can be controlled by insecticides; however, a more environmentally friendly and sustainable use of our agricultural resources demands a reduction of pesticide use. This requires breeding of resistant cultivars, which is facilitated by an understanding of the genetic control underlying resistances and potentially by genomics-assisted breeding. 

### 4.1. Phenotypic Variation of Cereal Leaf Beetle Resistance

The heritability obtained in this study was moderate with 0.63, but nevertheless acceptable considering that the phenotypic data stem from a single location and given the complexities of insect reproductive behavior [16]. One difficulty in evaluating insect resistance in the field under natural infestation is achieving an even infestation level throughout the test field. We found highly infested plots throughout the field, indicating that in general all plots were exposed to the pest. Weak trends of areas with generally higher or lower infestation were discernible, that could, however, be accounted for by spatial modeling (Figure S1). Taken together, the observed significant genotypic variance and the full range of cereal leaf beetle resistance in the investigated panel of 876 winter wheat cultivars illustrate the potential to improve this trait through breeding.

### 4.2. Plant Characteristics Affecting Cereal Leaf Beetle Resistance 

Interestingly, the analysis of cereal leaf beetle susceptibility dependent on the cultivars’ origin revealed differences between the countries. The higher average susceptibility observed for some countries thereby coincided with an on average earlier heading time of the cultivars from these countries [17,18]. We therefore analyzed the association between cereal leaf beetle susceptibility and heading time, which revealed a higher susceptibility of the earlier heading cultivars than the later heading ones (Figure 2). The entire life cycle of the cereal leaf beetle can take from 10 to 90 days, strongly depending on temperature, with an average of ~40–50 days under normal spring-time temperatures [2]. The adults emerge from overwintering in early spring, and then move into the crops and begin to lay eggs. The hatched larvae reach full size in 10–14 days at optimal temperature, and then drop to the soil surface to burrow down and pupate. The lower infestation of the later heading genotypes observed here may thus be caused by an avoidance of the pest and less by a direct resistance mechanism. Heading time ranged from 162 to 196 days after 01 January, thus covering approximately an entire month between the earliest and the latest heading cultivar. It thus appears likely that the flag leaves of the earlier heading genotypes were already available when the females started to deposit their eggs, while this period of oviposition may have passed when the flag leaves of later heading plants were developing, or was at least substantially shortened. Interestingly, Sherman et al. [19] reported a similar finding for infestation of a RIL population with the wheat stem sawfly, for which they observed a negative correlation of −0.57 between infestation and heading date. Our result thus underscores the effect of heading time, or more generally of the phenological development of the crop, on insect resistance. Not only is the life cycle of the insect strongly temperature-dependent, but so is wheat development, as evidenced by the requirement of the cultivars of this panel to reach a certain number of temperature degree days before heading is initiated [18,20]. It thus appears likely that the development of the pest and the crop advance more or less simultaneously, such that it is always the earlier heading genotypes that are more affected by the pest. The lower correlation observed between plant height and cereal leaf beetle resistance is likely due to the correlation between plant height and heading time (0.39, *p* < 0.001).

Initial cereal leaf beetle resistance in wheat has been associated with pubescence, i.e., leaf trichome density or length, possibly by deterring oviposition, and thus reducing egg populations [4,21]. Due to the difficulty in assessing this pubescence, we have not evaluated leaf hair traits, but have scored flag leaf glaucousness. We observed a significant correlation between cereal leaf beetle resistance and flag leaf glaucousness, with more glaucous leaves being less susceptible. This correlation was observed in early (*Ppd-D1a*) as well as later heading (*Ppd-D1b*) cultivars, and thus, does not appear to be an artifact, due to different levels of glaucousness in plants with different heading time. Sherman et al. [19] observed no significant correlation between wheat stem sawfly infestation and glaucousness in their RIL population, but in contrast to the cereal leaf beetle, the wheat stem sawfly attacks its host through the stem. Nevertheless, the association between cereal leaf beetle resistance and flag leaf glaucousness is less clear than the effect of heading time, and might also be an artifact, and consequently needs to be validated in independent populations. Taken together, our results show that plant characteristics such as heading time, or more general, developmental differences, can substantially affect cereal leaf beetle infestation in wheat.

### 4.3. Deciphering the Genetic Architecture Underlying Cereal Leaf Beetle Resistance

The genome-wide scan revealed a putative QTL on chromosome 2D, that through candidate gene analysis, was verified as *Ppd-D1* (Figure 3). This is in agreement with the observed strong effect of heading time on cereal leaf beetle resistance, and the fact that *Ppd-D1* is the major QTL underlying variation in heading time in this panel [18]. *Ppd-D1* was also found to be the major QTL of cereal leaf beetle resistance, explaining almost half of the genotypic variation. Earlier heading cultivars carrying the photoperiod insensitive *Ppd-D1a* allele showed an on average significantly higher susceptibility than the photoperiod sensitive *Ppd-D1b* plants (Figure 4a). 

Using the exploratory significance threshold, additional putative QTLs were identified on several chromosomes (Table 1). This includes a QTL on chromosome 5B, which was only present in the photoperiod sensitive *Ppd-D1b* group, and which is located in the same genomic region (579.1 Mbp, IWGSC RefSeq v1.0) as *Vrn-B1* (573.8 Mbp). Notably, the different *Vrn* and *Ppd* genes are known for their epistatic interactions [22]. Thus, while all genotypes in this panel carry the *Vrn-B1* winter allele, the QTL might be caused by allelic variation at the *Vrn-B1* locus, in line with the observed effect of developmental differences on cereal leaf beetle infestation. Most of the QTL must be regarded as small-effect QTL, with only little contribution to the genotypic variance of cereal leaf beetle resistance. However, three explained more than 4% of the genotypic variance, and while one of them might be *Vrn-B1*, two of these putative QTL did not coincide with QTL identified for heading time or flag leaf glaucousness in this panel of wheat cultivars [9,18]. These loci therefore warrant further research, as they may be involved in a resistance mechanism that will allow one to improve resistance without affecting agronomic traits or performance. The putative QTL identified here did not coincide with those reported previously by Joukhadar et al. [5] We did identify QTL on chromosomes 3B and 7D, but their positions are probably different from the reported ones, even though this is difficult to say, given that different marker systems were used, and they explained only a small proportion of genotypic variance. This corroborates the conclusion that resistance to cereal leaf beetle is complex and dependent on the genetic material underlying the study.

To complement our picture of the genetic architecture underlying cereal leaf beetle resistance in wheat, we employed genomic prediction. This approach uses genome-wide markers and allows us to also capture the effects of QTL that escape detection in association mapping [23,24]. The higher prediction accuracy obtained by this approach illustrates that the genotypic variance not explained by the identified putative QTL is at least in part attributable to additional additive genetic loci with small effects. In summary, the association mapping and genomic prediction approaches revealed a complex genetic control of cereal leaf beetle resistance in wheat, with *Ppd-D1* as a major QTL and numerous QTL with mostly small effects.

## 5. Conclusions

In this study, we investigated resistance to cereal leaf beetle in a large panel of winter wheat cultivars. We observed a strong impact of heading time on infestation, with earlier heading cultivars being more strongly affected, probably due to their leaves being available for oviposition for a longer and more optimal period of time. The photoperiod insensitive, early-heading allele *Ppd-D1a* is mainly employed in countries of lower latitude, as in southern Europe, in order to escape heat and drought stress during summer. An optimal timing of heading is essential for adaptation and maximization of the yield potential, and thus, is more or less fixed for a given target region. However, also within the two *Ppd-D1* allelic groups, the variation ranged from resistant to susceptible. This variation appears to be attributable to many QTLs with mainly small effects. Consequently, the potential for a marker-assisted selection based on an identified QTL appears low. Cereal leaf beetle resistance may thus best be improved by classical phenotypic selection in fields with natural infestation, or alternatively by genomic selection. The latter is, however, more expensive, and probably only worthwhile when marker profiles are available.

It has previously been suggested that the attraction of insects to different genotypes of their host may be controlled by the differential production of volatile compounds by the plants [25,26,27]. This was not investigated in the current study, but warrants further research also within the context of cereal leaf beetle resistance. Despite the efficiency of controlling this insect pest by insecticides, the breeding of resistant cultivars is an important component of an integrated pest management and sustainable agriculture. Collectively, our results add to the comparably small body of knowledge on the genetic architecture underlying agriculturally important insect pests.

## Figures and Tables

**Table 1:** Markers identified as significantly associated with cereal leaf beetle susceptibility in wheat.

Gene/Marker	Chr.	Pos. (cM)	Pos. (bp) ^a^	*P* Value	*p_G_*	Effect	*p* ^b^
*Ppd-D1*	2D	~51		2.7 × 10^−6^	35.3	0.46	0.81
D1104237	2D	244.8	568.757.425	1.57 × 10^−6^	6.2	0.43	0.87
Additional putative quantitative trait loci (QTL) ^c^							
D2255871	4A	236.8	720.048.127	5.17 × 10^−5^	2.4	0.15	0.09
S1100606	7A	46.4	34.920.176	1.47 × 10^−4^	0.6	0.34	0.88
D1208731	2B	76.7	65.468.079	5.17 × 10^−5^	2.6	0.13	0.85
D1062313	3B	28.7	13.410.285	3.07 × 10^−4^	0.7	−0.37	0.94
D1233649	5B	161.7	579.076.944	1.8 × 10^−5^	6.5	0.34	0.89
S1027735	6B	27.2	34.050.222	1.9 × 10^−4^	4.0	0.17	0.53
D977492	7B	188.8	688.130.100	3.2 × 10^−4^	2.5	−0.22	0.70
D1301286	3D	101.0	100.414.776	2.5 × 10^−4^	1.1	0.27	0.72
D1109181	6D	103.8	157.373.073	4.7 × 10^−4^	0.1	−0.32	0.79
D1129811	7D	188.5	207.189.065	3.5 × 10^−4^	1.5	0.28	0.85

^a^ Physical position in the wheat reference genome (IWGSC RefSeq v1.0). ^b^ Frequency of the allele increasing resistance. ^c^ Significant at the exploratory threshold of *p* < 0.0005.

## Supplementary Materials

The following are available online at https://www.mdpi.com/2223-7747/9/9/1117/s1, Table S1: Summary statistics for cereal leaf beetle (CLB) susceptibility, Table S2: Assessment of the putative QTL identified in the full data set in the photoperiod sensitive *Ppd-D1b* and photoperiod insensitive *Ppd-D1a* subsets, Figure S1: Cereal leaf beetle (CLB) infestation in the field, Figure S2: Results from location Hohenheim.
