# Supplementary material for: Genetic Architecture of Cereal Leaf Beetle Resistance in Wheat
Source: Plants (Basel). 2020 Aug 28;9(9):1117. doi: 10.3390/plants9091117 (PMC7570205; doi:10.3390/plants9091117)
Supplement: Supplementary file 1 [file plants-09-01117-s001.pdf]

Table S1. Summary statistics for cereal leaf beetle (CLB) susceptibility.

	CLB
Min	0.95
Mean	2.79
Max	5.26
σ_G^2	0.37**
σ_e^2	0.25
h^2	0.63

Genotypic variance (σ_G^2), error variance (σ_e^2), and heritability (h^2). ** significant at the 0.01 probability level.

Table S2. Assessment of the putative QTL identified in the full data set in the photoperiod sensitive *Ppd-D1b* and photoperiod insensitive *Ppd-D1a* subsets.

Gene/Marker	Chr.	Pos. (cM)	*Ppd-D1b* subset			*Ppd-D1a* subset		
			p_G	Effect	$p^{\$}$	p_G	Effect	$p^{\$}$
D1104237	2D	244.8	6.4	0.28	0.94	16.8	0.21	0.57
Additional putative QTL[&]								
D2255871	4A	236.8	3.3	0.16	0.09	2.0	0.12	0.10
S1100606	7A	46.4	0.7	0.18	0.93	2.3	0.18	0.66
D1208731	2B	76.7	2.4	0.12	0.85	12.5	0.32	0.89
D1062313	3B	28.7	0.2	-0.12	0.97	4.5	-0.28	0.83
D1233649	5B	161.7	12.6	0.35	0.91	0.3	0.08	0.80
S1027735	6B	27.2	5.7	0.16	0.53	1.8	0.08	0.44
D977492	7B	188.8	3.6	-0.16	0.75	1.3	-0.07	0.48
D1301286	3D	101.0	1.9	0.15	0.79	0.4	0.15	0.40
D1109181	6D	103.8	0.0	-0.15	0.88	1.3	-0.20	0.43
D1129811	7D	188.5	0.9	0.17	0.90	4.3	0.08	0.60

[&] Significant in the full data set at the exploratory threshold of $P < 0.0005$

[$] Frequency of the allele increasing resistance

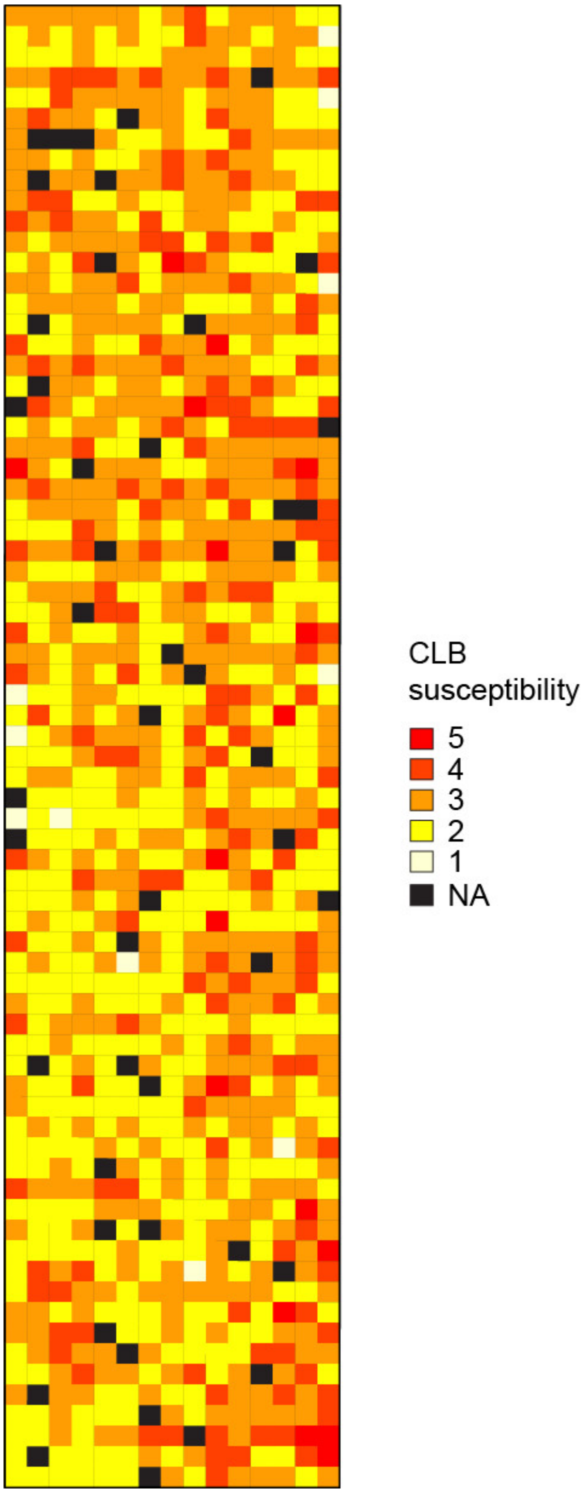

Figure S1. Cereal leaf beetle (CLB) infestation in the field. The squares represent the observation plots in the field.

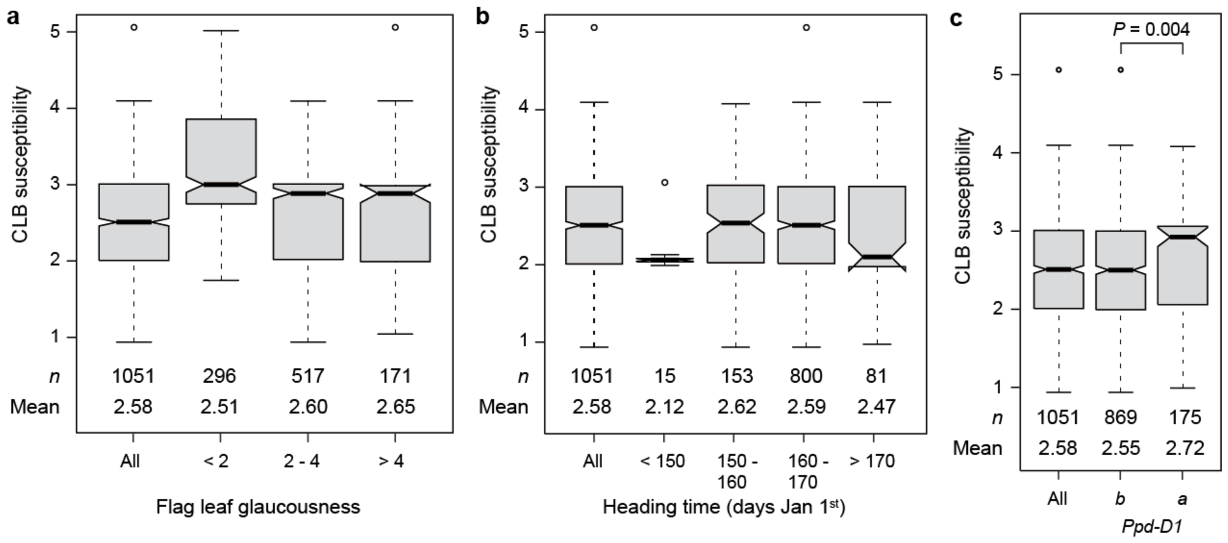

Figure S2. Results from the second location Hohenheim. Boxplots showing the association between cereal leaf beetle (CLB) susceptibility and (**a**) flag leaf glaucousness and (**b**) heading time. (**c**) Effect of the photoperiod regulator *Ppd-D1*.

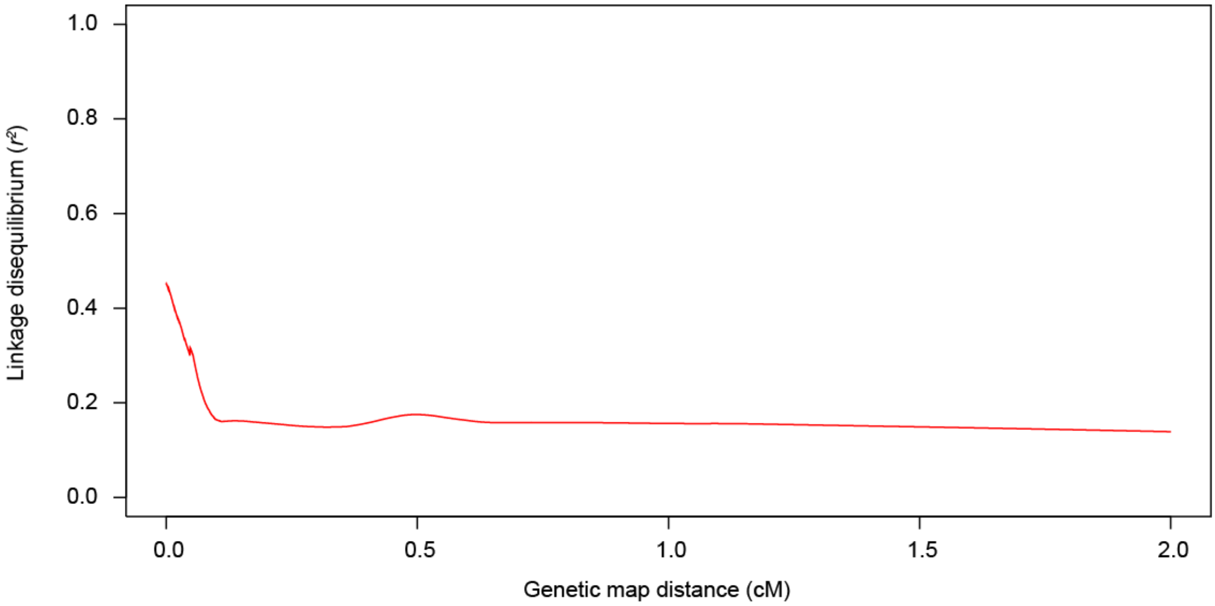

Figure S3. Decay of linkage disequilibrium with genetic map distance. The rather rapid average decay of linkage disequilibrium illustrates the on average high mapping resolution that can be realized in this panel.
